# Case of a Nail Re-Produced after Amputation of the First Finger-Joint

**Published:** 1816-07

**Authors:** 


*For the London Medical and Physical Journal.*

### *Case of a Nail re-produced after Amputation of the First Finger-Joint;* by W. S.

OBSERVING that you have lately made some remarks on the reproduction of lost parts in the human body, I beg leave to communicate the following case, the particulars of which I can state with much confidence, as I had an opportunity of giving it an attentive examination, and the subject of it is an intelligent and respectable person.

A. G. a tradesman of Edinburgh, met with an accident, by which the extremity of the fore-finger of his right hand was severely crushed and lacerated. Having been neglected for more than a week, and exposed to fresh injury, violent inflammation inflammation supervened, attended by extreme tumefaction, and followed by gangrene; in consequence of which the last phalanx was amputated at the joint.

The skin of the finger subsequently sloughed off as far as the knuckle, a portion of loose tendon was cut away on each side, the swelling subsided, and the part gradually healed; soon after which, a horny substance was formed at the extremity of the stump.—I cannot accurately state the age of this person at the time of the accident, but believe he was upwards of twenty. I saw him about twenty-five years afterwards, at which time the remains of the finger were diminished in size, and the joint between the first and second phalanxes anchylosed.

The new nail is a dense laminated substance, resembling the claw of an animal; it issues from the end of the stump, and bends abruptly round towards its back, terminating in an obtuse point, which rests on the skin. It serves as an useful defence to the mutilated part, and has a slow growth requiring to be cut four or five times in a year.

12, *Great Castle-street, Cavendish-square;*
*May* 15, 1816.

